# Well-being at work, productivity, and coping with stress during the COVID-19 pandemic

**DOI:** 10.47626/2237-6089-2021-0250

**Published:** 2022-11-09

**Authors:** Bibiana Bolten Lucion Loreto, Sofia Cid de Azevedo, Antonio Geraldo da Silva, Leandro Fernandes Malloy-Diniz, Felipe Ornell, Letícia Maria Akel Mameri Trés, Félix Henrique Paim Kessler, Melina Nogueira de Castro

**Affiliations:** 1 Serviço de Psiquiatria Hospital de Clínicas de Porto Alegre Universidade Federal do Rio Grande do Sul Porto Alegre RS Brazil Serviço de Psiquiatria, Hospital de Clínicas de Porto Alegre (HCPA), Universidade Federal do Rio Grande do Sul (UFRGS), Porto Alegre, RS, Brazil.; 2 Universidade do Porto Porto Portugal Universidade do Porto, Porto, Portugal.; 3 Associação Brasileira de Psiquiatria Rio de Janeiro RJ Brazil Associação Brasileira de Psiquiatria (ABP), Rio de Janeiro, RJ, Brazil.; 4 Asociación Psiquiátrica de América Latina Brasília DF Brazil Asociación Psiquiátrica de América Latina (APAL), Brasília, DF, Brazil.; 5 Universidade Federal de Minas Gerais Belo Horizonte MG Brazil Universidade Federal de Minas Gerais (UFMG), Belo Horizonte, MG, Brazil.; 6 Fundação Mineira de Educação e Cultura Belo Horizonte MG Brazil Fundação Mineira de Educação e Cultura, Belo Horizonte, MG, Brazil.; 7 Associação Brasileira de Impulsividade e Patologia Dual Brasília DF Brazil Associação Brasileira de Impulsividade e Patologia Dual, Brasília, DF, Brazil.; 8 Centro de Pesquisa em Álcool e Drogas HCPA UFRGS Porto Alegre RS Brazil Centro de Pesquisa em Álcool e Drogas, HCPA, UFRGS, Porto Alegre, RS, Brazil.; 9 Departamento de Psiquiatria e Medicina Legal Faculdade de Medicina UFRGS Porto Alegre RS Brazil Programa de Pós-Graduação em Psiquiatria e Ciências do Comportamento, Departamento de Psiquiatria e Medicina Legal, Faculdade de Medicina, UFRGS, Porto Alegre, RS, Brazil.; 10 Serviço de Psiquiatria de Adições e Forense HCPA UFRGS Porto Alegre RS Brazil Serviço de Psiquiatria de Adições e Forense, HCPA, UFRGS, Porto Alegre, RS, Brazil.

**Keywords:** COVID-19, work, mental health, occupational health and safety, coping, stress

## Abstract

This study aims to analyze the mechanisms through which the coronavirus disease (COVID-19) pandemic impacts on well-being at work and on productivity. The secondary objective is to identify stress management strategies for the work environment during the pandemic. This is an integrative review. Phase 1 consisted of searches of open access electronic databases (MEDLINE, SciELO, Bireme, and LILACS) for papers published in 2020 addressing mental health, work, and pandemics. Phase 2 consisted of selecting literature recommended by specialists in occupational psychiatry and positive psychology. These materials were read and critically analyzed. Forty references were included in the literature review. The articles reviewed were classified into the following categories: articles concerning work relationships in Brazil; articles describing the impact of pandemics on mental health and work; articles focusing on the work of health professionals during pandemics; articles about well-being at work; and papers proposing strategies to improve well-being and productivity and to promote mental health. The COVID-19 pandemic can have a significant impact on workers’ mental health and productivity. Most professionals face a need to adapt to changes, which can decrease their feeling of well-being. Consequently, strategies to promote well-being and mental health in the work environment should be a priority. Work routines were modified after the COVID-19 pandemic set in and assessing these changes is essential to maintain workers’ mental health. By so doing, it is possible to promote general well-being and post-traumatic recovery and reduce stress levels.

## Introduction

Coronavirus disease (COVID-19) was declared a pandemic by the World Health Organization (WHO) in March 2020 and since then it has caused widespread changes around the world.^1^ Besides the direct harmful effects of the new coronavirus, some groups seem to be more vulnerable to psychiatric symptomatology, stress, and burnout, which are factors that usually lead to a diminished quality of life.^2^ Another concern already expressed by some affected countries relates to the economic impact of the pandemic, since many sectors have been undermined at varying degrees of intensity.^3^ In this context, people need to adapt to new and different routines of life and professional activities, while society keeps demanding high productivity. As stated previously, the COVID-19 pandemic poses new occupational health challenges involving, for example, risk of contamination during work activities of people whose activities are performed in proximity to other people. In these cases, occupational demands impose additional distress by preventing workers from following recommendations concerning social distancing and increasing their risk of contagion. In other cases, there is the great challenge of having to adapt to a new framework of activities including changes in work routines and environment.^4^

In Brazil, different types of work are classified according to their contracts, legality, and formality. Each type is impacted in a different way. Therefore, the application of the concept of well-being at work also has many differences, as do impacts on workers’ mental health.^5^

Formal employment in Brazil consists of hiring in compliance with labor laws that guarantee employees a greater feeling of security with regard to salary and stability. Formal jobs can be divided into administrative and operational roles. Administrative services can be adapted to modalities such as home working or telework, which is necessary to keep the economy functioning while organizations struggle to maintain business continuity during the pandemic. However, the home office model being implemented does not offer the same possibilities as the format used before the pandemic. The current situation can cause adaptation problems, since workers may not have an appropriate environment to perform their work duties. When working at home, possibly among other people and domestic tasks, the boundaries between work and personal life can become blurred, with negative consequences for workers’ well-being.^6^

In turn, adaptation to the remote working model is not always possible for operational services. As a result, workers whose functions constitute essential activities must keep performing their roles on site and thus need to follow all the biosafety rules. This situation can increase their levels of stress and trigger internal conflicts within teams because in many scenarios only part of the team is authorized to work remotely.^7^ An observational study conducted recently in Chinese provinces affected by COVID-19 suggests that, despite the risks, those who keep their work routine unchanged have better levels of mental health than those allocated to remote work (home office).^8^ This highlights the importance of work both for the mental health of the population and for the economic recovery of a country or region.

In this context, with such diverse measures affecting the work teams, it can be challenging to maintain a healthy work organization. The problem is exacerbated for formal workers who perform non-essential services, because the operational workers in these areas are usually affected by unemployment due to closure of some companies for indefinite periods. The literature shows that the current crisis had already caused a significant reduction in economic activity and hours worked in the first six months of 2020.^9^ This leads to aggravation of existing mental pathologies and development of others, including a possible increase in suicide rates.^10^

Informal work is in an opposite situation. Informal employees are not registered with social security or tax authorities via the Brazilian “carteira de trabalho” system, do not therefore receive benefits, and are subject to more flexible work schedules.^9^ Informal workers also remain in the labor market because they do not have any legal guarantees to maintain them financially if they cease work activities. It is possible that informal workers endure increased levels of stress and work for more hours than recommended to meet the increased demands for some essential services during the pandemic.^11^ Many informal workers also have difficulty in complying with social distancing measures because they do not have appropriate social security protection to cover periods of crisis, meaning they must keep working to maintain an income. Moreover, the economic recession caused by the pandemic can increase rates of unemployment in this sector, posing an additional threat to well-being, since these people do not have the guaranteed benefits of formal work. The COVID-19 pandemic can therefore negatively impact workers in the informal sector and aggravate inequalities in all these ways.^12^

Another category, autonomous workers and independent professionals, are defined as people who provide services on their own and guarantee their income without any kind of employment relationship (whether formal or informal). Medical workers in particular faced urgent implementation of telemedicine to minimize exposure of the population without severe diseases to health services, while maintaining clinical care in different specialties. This new situation for many doctors is considered a stress factor because it demands a minimum level of mastery of technology and personal readiness to accept new forms of working. In addition, telemedicine also involves other barriers, such as difficulties for patients to access technology, which can limit the medical care provided by independent professionals. Issues like these affect professionals’ well-being and they may question the quality of the service they are providing to their patients.^13^

Another specific way of providing services, volunteer work, is characterized by working without any kind of remuneration. This type of work has been of great relevance in the pandemic. The feeling of well-being afforded by these activities is significant and is linked to the affective component of this type of work. Performing volunteer work protects mental health, even when social distancing is not observed and it involves increased exposure to pathogenic risks. Workers report positive experiences with volunteer work, like strengthening of the notion of purpose and well-being derived from the impact of these activities in the community. Although the pandemic has imposed changes to work models, the current situation can be favorable to the practice of volunteerism in several sectors.^14^

The last type of work is that performed by entrepreneurs who provide services to their own companies and obtain their income from this source. The current situation of the country in recent months forced many businessmen to cease their activities and compelled them to dismiss their employees. Consequently, it is possible that, with no concrete prospect of improvement in the economic scenario, they lose the sensation of well-being at work and part of their meaning of life, giving rise to hopelessness and helplessness.^10^

The exact impacts of COVID-19 on mental health are still unknown, but undoubtedly tend to be significant in at least part of the population. There are many factors involved, like social distancing, generalized fear, and grieving without the possibility of a final farewell. In periods of crisis, insecurity about employment stability and financial concerns usually become evident.^11^ Along the same lines, the need for social distancing because of the pandemic isolates workers from the social support found in work relations, which is a significant protective factor against stress.^15^ The support derived from the work environment includes interaction with colleagues and leaders and can be imparted through practical assistance, encouragement, emotional support, and other ways, which are associated with greater satisfaction at work.^16^ Workers with special needs and those deprived of this support network for prolonged periods report greater harm to their well-being at work and their productivity.^17^ It is plausible that drastic changes to routines, compounded by the impact on mental health and economic instability, affect workers’ ability to cope with the demands of work and, in the long term, hamper economic recovery of organizations and the country as a whole.^4^

It is important to understand the relation between individuals and their work environment. Well-being at work positively influences workers’ motivation, their performance, and their effort. It also improves organizational behavior and interpersonal relationships. On the other hand, low levels of well-being at work can negatively impact organizations.^18^ During a pandemic, acting in a positive way is a great challenge, since the factors involved in well-being at work are greatly weakened. However, during this phase of adaptation, there may also be opportunities to reassess types of work and forms of working and take measures to achieve a better quality of well-being at work. This study aims to analyze the mechanisms through which the COVID-19 pandemic impacts on well-being at work and on productivity. The secondary objective was to identify stress management strategies during the pandemic in the context of work.

## Methods

This is an integrative review of both quantitative and qualitative studies addressing the subjects of interest. In Phase 1 of the review presented in this paper, conducted from May to June of 2020, authors SCA and BBLL conducted bibliographic searches to identify primary, descriptive, exploratory, qualitative, and quantitative studies published in 2020 and addressing mental health in the work environment during the pandemic. The following descriptors were used to search for articles: “Bem-estar no trabalho,” “Bem-estar e pandemia,” “Well-being at work,” “Work and Covid-19,” “Work and pandemic,” “Mental health and well-being at work,” “Mental health and pandemic.” The same search was performed on each of the following open access databases: MEDLINE, SciELO, Bireme, and LILACS. The inclusion criteria were articles on workers’ mental health and articles describing the impact of pandemics on mental health and work, with any study design. Articles that were not written in Portuguese or English, articles focusing only on the physical health of workers, articles on informal work, and articles involving work of minors were excluded. Phase 2 of this review was conducted from July to November of 2020, and consisted of reviewing articles with any study design, including grey literature, recommended independently by specialists in occupational psychiatry (LMAMT and AGS) and positive psychology (PP) (MNC and LFM-D). Phase 3 was performed by all authors of the paper and consisted of reading, categorizing, and critically analyzing the material compiled in Phases 1 and 2, producing the results and discussion presented below.

## Results

The literature review resulted in inclusion of 40 references. They can be categorized as 12 cross-sectional studies, 10 literature reviews, six editorials, three book chapters, three clinical trials, two meta-analyses, two cohort studies, one systematic review, and one guidance document concerning the pandemic. These materials were published between the years of 1996 and 2020. The articles reviewed were also classified into the following categories: articles concerning work relationships in Brazil; articles describing the impact of pandemics on mental health and work; articles focusing on the work of health professionals during pandemics; articles about well-being at work; and papers proposing strategies to improve well-being and productivity and promote mental health. Phase 1 of the bibliographic search included three editorials and one literature review. Phase 2 yielded 36 articles recommended by specialists in occupational psychiatry and positive psychiatry, comprising 12 cross-sectional studies, two longitudinal studies, three editorials, nine literature reviews, three book chapters, one guidance document, two meta-analyses, one systematic review, and three clinical trials. The results of Phase 3, which consisted of categorizing and analyzing these articles, are summarized in Table 1.

**Table 1:** Characteristics of the materials included in the review

Articles	Year of publication	Type	Phase of the search
Lai et al.^19^	2019	Cross-sectional study	Phase 2: specialist recommendation (LMAMT)
Shultz et al.^20^	2010	Longitudinal study	Phase 2: specialist recommendation (AGS)
Ornell et al.^21^	2020	Editorial	Phase 2: specialist recommendation (AGS)
Da Silva et al.^22^	2020	Editorial	Phase 2: specialist recommendation (AGS)
Hamouche^23^	2020	Literature review	Phase 1: database review (SCA/BBLL)
Schaufeli & Salanova^24^	2014	Book chapter	Phase 2: specialist recommendation (MNC)
Paschoal et al.^25^	2013	Literature review	Phase 2: specialist recommendation (LMAMT)
Van Beek et al.^26^	2012	Cross-sectional study	Phase 2: specialist recommendation (MNC)
Warr^27^	2006	Literature review	Phase 2: specialist recommendation (AGS)
Jung & Yoon^28^	2014	Cross-sectional study	Phase 2: specialist recommendation (LMAMT)
Kelloway et al.^29^	2013	Cross-sectional study	Phase 2: specialist recommendation (MNC)
O’Reilly et al.^30^	2020	Guidance document	Phase 2: specialist recommendation (AGS)
Steel et al.^31^	2018	Meta-analysis	Phase 2: specialist recommendation (LMAMT)
Noronha^32^	2000	Literature review	Phase 2: specialist recommendation (AGS)
Lee & Ashfort^33^	1996	Meta-analysis	Phase 2: specialist recommendation (LFM-D)
Mostert & Rothmann^34^	2006	Cross-sectional study	Phase 2: specialist recommendation (LFM-D)
Santos & Ceballos^35^	2013	Literature review	Phase 2: specialist recommendation (LMAMT)
Stansfeld^36^	2005	Book chapter	Phase 2: specialist recommendation (LFM-D)
Niedhammer & Chea^37^	2003	Cross-sectional study	Phase 2: specialist recommendation (LFM-D)
Schutte et al.^38^	2014	Cross-sectional study	Phase 2: specialist recommendation (LMAMT)
Wolter et al.^39^	2019	Cross-sectional study	Phase 2: specialist recommendation (LMAMT)
Oliveira & Sousa^40^	2018	Literature review	Phase 2: specialist recommendation (LFM-D)
Makikangas & Kinnunen^41^	2003	Longitudinal study	Phase 2: specialist recommendation (LFM-D)
Sonnentag^42^	2015	Literature review	Phase 2: specialist recommendation (LMAMT)
Van Horn et al.^43^	2004	Cross-sectional study	Phase 2: specialist recommendation (LMAMT)
Melo et al.^44^	2016	Systematic review	Phase 2: specialist recommendation (LMAMT)
Folkman & Moskowitz^45^	2004	Literature review	Phase 2: specialist recommendation (LMAMT)
Rocha-Sobrinho & Porto^46^	2012	Cross-sectional study	Phase 2: specialist recommendation (LMAMT)
Hirschle et al.^47^	2019	Cross-sectional study	Phase 2: specialist recommendation (LMAMT)
Tugade & Fredrickson^48^	2007	Literature review	Phase 2: specialist recommendation (LFM-D)
Shah et al.^49^	2020	Editorial	Phase 1: database review (SCA/BBLL)
Fessel & Cherniss^50^	2020	Editorial	Phase 1: database review (SCA/BBLL)
Rajkumar^51^	2020	Editorial	Phase 1: database review (SCA/BBLL)
Ghosh^52^	2018	Literature review	Phase 2: specialist recommendation (MNC)
Coo & Salanova^53^	2018	Clinical trial	Phase 2: specialist recommendation (MNC)
Vazquez & Schaufeli^54^	2019	Book chapter	Phase 2: specialist recommendation (MNC)
Ouweneel et al.^55^	2013	Clinical trial	Phase 2: specialist recommendation (MNC)
Peñalver et al.^56^	2019	Clinical trial	Phase 2: specialist recommendation (MNC)
Tombaugh^57^	2005	Editorial	Phase 2: specialist recommendation (MNC)
Muceldili et al.^58^	2013	Cross-sectional study	Phase 2: specialist recommendation (MNC)

## Discussion

### Work in Brazil and the impact caused by the pandemic

Evidence shows that excessive work demands are related to increases in absenteeism, self-reported health problems, mental disorders like depression and anxiety, burnout, coronary disease, and musculoskeletal complaints.^59-61^ Moreover, work overload (including monotonous and meaningless assignments) can increase work-related stress and can have negative effects on health, well-being, and job satisfaction.^62^

During the COVID-19 pandemic, health workers on the frontline need to work under pressure, for longer hours, in consecutive shifts, with extra workload and reduced periods of rest. Other professionals, like those involved in production, delivery, and transportation of essential utilities and those responsible for the safety and protection of the population, face similar situations in terms of work regime. Even professionals working in home office settings can feel an overload of assignments.^63^

Another relevant point is the economic impact of quarantine and the fear of unemployment. The costs of quarantine during the severe acute respiratory syndrome (SARS) epidemic of 2004 have already been the object of research interest, in a study comparing the economic costs and the benefits associated with this public health intervention. Despite the results indicating that quarantine reduced economic losses, it is difficult to quantify the psychological impact on workers who feared for their health and worried about economic instability.^64^ It can be inferred that the levels of stress to which employees in many different sectors are exposed in such an unstable context is higher than usual. Consequently, people can become less productive and even more vulnerable to severe psychological symptoms.^65^

### The work of healthcare professionals

Professionals directly or indirectly involved in healthcare during the pandemic can be classified by their different types of work. Regardless of their mode of working, these people are on the front line of fighting the crisis. In this critical situation, health professionals directly involved in diagnosis and treatment of patients with COVID-19 are at greater risk of developing psychological distress and other symptoms of deteriorating mental health.^19^ The growing number of confirmed and suspected cases, excessive workload, depletion of personal protective equipment, ubiquitous media coverage, lack of specific medicines, and feelings of lack of support are some of the psychosocial risk factors these workers face.^20^ In the specific case of COVID-19, the high transmissibility of the disease also directly affects human relationships and intensifies professionals’ feelings of insecurity. There is the fear of autoinoculation while caring for patients and the possibility of transmitting the virus to families and colleagues. These factors also put this group at higher risk of developing dysphoric emotional states.^21^

Studies related to the SARS epidemic of 2003 revealed adverse psychological reactions among healthcare professionals. Fear of contagion, uncertainty, stigmatization, fear of going to work, desire to resign, high levels of stress, anxiety, and depression were all observed and could have long term psychological consequences.^22^ The trend is that these findings may be replicated in the current pandemic, since health professionals on the front line are already reporting higher levels of anxiety and lower rates of well-being.^23^

Some healthcare professionals at higher risk of contagion by coronavirus were advised to keep social distance even from their families, which can intensify the perception of stress and aggravate emotional difficulties. This group requires special attention and care, so development of concepts of well-being at work is essential to minimize the negative impacts caused by the current scenario. In addition to these professionals on the front line, other sectors may also be suffering from issues associated with work or the lack of it, since boredom is also related to burnout.^24^

### Well-being at work

Well-being at work has traditionally been conceptualized according to two different approaches. The first is as a manifestation of affective evaluation of the characteristics of the job, whose central point would be a balance between positive and negative evaluations made by the individual concerned. If the balance is positive, well-being is evident; if negative, its absence is observed.^25^ This first approach comes close to the concept of subjective well-being. The second approach defines well-being at work as a set of positive evaluations about its aspects, such as motivation, setting, and remuneration, among others.^26^

Although affect is a central dimension of the concept of occupational well-being, it can be understood as the positive evaluation of various dimensions of work, specifically affective, professional, social, cognitive, and psychosomatic. However, there are three other factors that must be considered when conceiving occupational well-being: (a) the cognitive process used to interpret the situation; (b) other people’s opinions; and (c) personality traits.^27^

### Well-being at work during the pandemic

When we think about well-being at work constructs and the situations that workers in general are subjected to in this pandemic scenario, it is evident that there is great loss in achievement of general well-being and especially of well-being at work. Most professionals currently face a need to adapt to change, which can constitute a risk factor and decrease subjective well-being.^28^ For some workers, the negative aspects of work may, for some time, supplant the positive ones, and we need to pay attention to this matter. With the possibility of workers presenting depressive and anxious symptoms, possibly triggered by the pandemic, adaptation to a new work system is likely to be necessary, especially with regard to the style of leaders, who are known to have an impact on workers’ well-being when they adopt a positive style, focused on the employee’s potential.^29^

The COVID-19 pandemic is impacting society as a whole and will have particular effects on individuals’ mental health. Consequently, work behavior and work relationships can change. Companies must pay attention to this dynamic scenario and seek to promote a healthy work environment, whether physical or virtual.^30^ To achieve this, it is worth remembering that some psychosocial factors can be targeted as potential protectors to promote workers’ health and well-being, leading to happiness and flourishing.^31^ A positive movement can be initiated by directing interventions towards aspects that boost motivation, productive behavior, and adequate performance, not only for productivity, but also for workers’ health.^32^

As part of the well-being at work construct, the specialized literature cites items such as demographic variables, social climate, personality traits, and coping strategies (coping, emotional regulation, mastery of the work environment, and autonomy). It is necessary to intervene in each item to minimize the negative impact and amplify the positive impact, seeking to increase well-being at work during the pandemic, as well as to develop other strategies that aim to reduce perceived stress.^33^

Demographic variables have a weak correlation with well-being at work, but it is important that companies pay attention to the conditions under which the worker will perform remote work. Assessment of a professional’s psychosocial reality must be carried out individually, proposing adjustments when necessary.^34^ In some situations, it may even be necessary to assist with the physical organization of the workplace, including provision of furniture, equipment, and platforms for performing work in an appropriate manner.

Social climate is the strongest predictor of well-being at work and is perhaps the element most affected at this time, since feelings of insecurity and helplessness in the face of the pandemic and quarantine are prevalent.^35^ The overload of negative and fake news contributes to worsen this situation. With social distancing, the individual’s relational field can be impaired.^36^ It is therefore important to avoid emotional distance, including between members of work teams, holding meetings virtually or by telephone.^37^

Still, in relation to the social climate, leaders have great significance, since they influence the environments and structures of work relationships. The leader’s posture, for example, can model the team’s behavior in this time of uncertainty. Leaders must act in a manner consistent with the recommendations made to teams and must adopt current policies, such as practicing social distancing and wearing protective masks.^38^ Literature also suggests an association between leaders with positive behavior and positive employee affect. Therefore, the pattern of leadership can affect the organization and employees’ well-being in different ways.^29^

Another important issue for improvement of the social climate by leaders is encouragement of the team’s autonomy, as well as creativity and acknowledgement.^40^ The possibilities for developing autonomy are greater, mainly due to the adaptation to working from home. Likewise, creative thinking is of the utmost importance, and can offer indispensable solutions at that time.

Personality traits are the third item of the well-being at work construct. These are innate characteristics of each individual that can be worked on positively, or negatively, and can be exacerbated in situations of intense social commotion, such as those we are currently experiencing. Job opportunities can link an employee’s characteristics and values to the purposes of their job functions.^41^ In addition to the effect of identifying meaning in the professional task, acknowledgement from managers and, in the current context, from society, of the professionals who are maintaining the country’s economy and of professionals who are intensely exposed to the coronavirus, is of paramount importance.^42^ It is essential that individuals from all work areas are able to recognize how much their jobs are contributing to society, so that even workers who previously did not feel a purpose in life at work can start to develop one.^43^

Dealing with negative emotions is a crucial job demand, especially for healthcare workers. Resources present in employment, such as autonomy, social support, or reward, but also personal resources, such as emotional regulation strategies, can reduce stress at work and increase well-being at work.^44^ Coping or coping strategies, which are also an item within the well-being at work construct, are cognitive and behavioral efforts to deal with situations of damage, internal and external demands of the relationship between the individual and the environment, or threats or challenges when a routine is not available.^45^ People are not always prepared to avoid stress, but the way they deal with it makes a difference to their mental health. Studies have demonstrated the power of coping as a predictor of occupational health, showing that the coping strategies individuals use are decisive in the evolution of stress and in development of occupational diseases. Coping also appears as a factor with a powerful impact on well-being at work.^46^

Emotional regulation (ER) is a mechanism through which individuals influence and control the way they express and experience emotions, involving use of strategies that may be more or less effective, with different impacts on emotional experience, behaviors, and physiological states.^47^ Emotional regulation has therefore been shown to be an important variable in individuals’ adaptation to stressors, minimizing the impact of stress on general well-being and providing a means for developing resilience in times of stress.^48^

### Proposed strategies

In recent months, literature has already been produced presenting reflections seeking to mitigate the impact of the pandemic. Flexible goals, transparent communication, psychological support, and provision of personal protective equipment (PPE) when necessary are some ways to prevent development of negative consequences, such as burnout.^49^ Other strategies can help to maintain the mental health of people in isolation, especially those who are under greater pressure and more susceptible to stress. Use of the abdominal breathing technique during moments of anxiety and insomnia, performance of mindfulness techniques at moments such as hand hygiene, and the practice of naming feelings are simple tasks that can have a significant impact on people at greater risk of illness.^50^ Initiatives to curb the spread of false information and educational campaigns to reduce stigma can also be ways to reduce this impact.^51^

Some specific interventions that were already gaining ground in different contexts can also prove effective in the current scenario. Some strategies to promote workplace happiness should be encouraged, like practicing optimism and gratitude, stimulating creativity, and maintaining some kind of social interaction.^52^ Mindfulness-based interventions have already been studied in the organizational environment, considering their influence on workers’ engagement, performance, and happiness. A controlled study published in 2018 compared the effects of mindfulness-based interventions on a small population of hospital employees with a control group. The results showed significant benefits, with moderate effect size, for levels of happiness, engagement, and performance at work.^53^

Interventions in the field of psychiatry and PP also deserve attention, especially since there are already studies carried out with online interventions, which are particularly necessary during social distancing.^54^ A survey that evaluated the effects of an individual online PP intervention focused on positive emotions, self-efficacy, and engagement at work. A total of 86 participants in the intervention group completed the study, which showed a significant impact on positive emotions and self-efficacy. Analysis of the results also showed that participants with low scores on the scales that measured positive emotions, self-efficacy, and engagement before the intervention benefited more than those who already had higher scores. These data reinforce the need to be attentive to employees with lower levels of engagement and well-being, who may be those that most benefit from these interventions.^55^

Although the topic of productivity in the organizational context and its relationship with affective aspects is still scarcely covered in the literature, some studies have already identified factors of relevance to individuals’ performance at work. A study that conducted two analyses with independent samples sought to demonstrate the mediating role the group’s social resources played between the group’s positive affect and performance. One of the analyses grouped 449 individuals in 112 small groups, performing a simulated creative task. The other was a field study that gathered scores from 2,159 employees grouped into 417 groups. In both studies, it was observed that the group’s social resources play a mediating role between the group’s positive emotions and their performance. These findings corroborate the need to bear in mind the importance of positive emotions and social resources in companies as a way to optimize productivity.^56^

However, the search for performance improvements should not depend only on interventions targeting employees. Positive leadership influences the organizational culture and can, consequently, stimulate productivity.^57^ Certain leadership patterns can be crucial in the current situation to encourage the search for creative and innovative solutions in the midst of the crisis. A study carried out in Turkey used questionnaires to evaluate the relationship between authentic leadership (AL) and the innovation capacity of 142 employees from different sectors. AL was shown to have positive correlations with employee creativity and innovation capacity.^58^

This literature review led to practical conclusions on which actions should be most beneficial for workers’ mental health in times of pandemic. Table 2 lists some coping strategies for promoting mental health, increasing well-being at work, and increasing productivity.

**Table 2:** Coping strategies for increasing well-being at work and promoting mental health

Individual strategies to control psychological symptoms
Diaphragmatic breathing
Mindfulness
Positive psychology techniques
Naming feelings
Listing reasons to be grateful
Paying attention to mood swings and anxiety and talking about those feelings
Developing a sense of collectivity in relation to the pandemic
Maintaining continuous medication use
Limiting exposure to pandemic-related news
Strategies for companies
Flexibility of goals
Reassessment of workload
Meeting optimization
Transparent communication
Stimulating creativity
Encouraging collaboration
Maintaining the culture of teamwork through virtual connections
Psychological support
Supply of personal protective equipment
Stimulating prevention and health promotion
Valuing the work of professionals exposed to risks
Strategies for home office
Taking care of ergonomics, properly positioning the computer monitor, mouse, and keyboard
Adequate ambient lighting
Keeping frequently used documents and objects close to hand
Avoiding working at night
Establishing work start and end times
Establishing a meal break
Taking short breaks (5-10 min) every hour of work, for stretching and relaxation
Healthy lifestyle
Balanced diet
Avoiding increased alcohol consumption and use of other drugs
Regular physical activity
Sleep hygiene
Restriction of exposure time to screens
Leisure time
Maintaining support network by virtual means

## Conclusion

It is well known that work routines were modified after the COVID-19 pandemic set in. Consequently, employees working in a variety of sectors may be subjected to work overload, stress, and psychological symptoms. The faster the process of assessing these changes in routines, the more likely it is that the workers will be able to exercise control over the environments in which they find themselves. Also, as adaptations are implemented, the feeling of well-being and the possibility for professionals to exercise their autonomy in the work environment will increase. Since research on this topic has yet to be published, an integrative review including all types of publications can provide a source of answers for very pressing questions.

In this sense, the prevalence of positive emotions at work and the perception that individuals can express and develop their potentials and skills and advance towards achieving their life goals, even in a context of adversity, tends to increase well-being at work during the pandemic. It is thus possible to promote general well-being and post-traumatic recovery and decrease the level of perceived stress. In addition, strategies to prevent mental illness should be developed to minimize damage and reduce factors associated with suffering and illness in the workplace.
